# The efficacy of laparoscopic presacral neurectomy in dysmenorrhea: is it related to the amount of excised neural tissue?

**DOI:** 10.4274/tjod.56588

**Published:** 2017-12-30

**Authors:** Murat Api, Ayşen Boza, Mehmet Ceyhan, Ecmel Kaygusuz, Hülya Yavuz, Olus Api

**Affiliations:** 1 İstanbul Medipol University Faculty of Medicine, Department of Obstetrics and Gynecology, İstanbul, Turkey; 2 American Hospital, Women’s Health Centre and Assited Reproduction Unit, İstanbul, Turkey; 3 University of Health Sciences, Zeynep Kamil Women and Children Diseases Training and Research Hospital, Clinic of Obstetrics and Gynecology, İstanbul, Turkey; 4 University of Health Sciences, Zeynep Kamil Women and Children Diseases Training and Research Hospital, Clinic of Pathology, İstanbul, Turkey

**Keywords:** Dysmenorrhea, nerve fiber, presacral neurectomy, laparoscopy

## Abstract

**Objective:**

To assess the correlation between the number of excised neural fibers and degree of pain relief following laparoscopic presacral neurectomy (LPSN).

**Materials and Methods:**

In this before and after study, 20 patients with severe midline dysmenorrhea [Visual Analogue Scale (VAS) >80 mm] unresponsive to medical therapy were consecutively enrolled. All patients underwent LPSN. The superior hypogastric plexus was excised and sent for histologic confirmation. Two pathologists counted the number of neural fibers in the surgically removed tissue. VAS was used for pain assessment before and 2nd, 3rd, 6th, and 12th months after the operations.

**Results:**

Out of the initial 20 patients undergoing LPSN, eight were excluded from the final analysis due to intraoperative diagnosis of endometriosis; therefore, the remaining 12 patients were evaluated. The pain scores significantly decreased at each follow-up visit compared with the preoperative period (p=0.002). The pathologists, who were blinded, reported the median (minimum-maximum) neural fiber count as 46 (20-85) and 47 (18-83). No significant correlation was demonstrated between the number of excised neural fibers and the amount of pain relief following LPSN.

**Conclusion:**

LPSN is an effective surgical procedure to control primary dysmenorrhea. Our preliminary results revealed that the degree of pain relief in cases of severe midline dysmenorrhea was not related to the amount of excised neural tissue in LPSN.

## PRECIS:

The degree of pain relief after laparoscopic presacral neurectomy is not related to the amount of excised neural tissue.

## INTRODUCTION

Dysmenorrhea, painful menstrual cramps, is a very common gynecologic problem with a prevalence of 43-90% in women of reproductive age^(1,2)^. Dysmenorrhea is such a challenge that it interferes with the performance of daily activities, and may even lead to job or school absenteeism. Therefore, the main goal of treatment is to provide pain attenuation sufficient to sustain the woman’s daily performance. Medical therapy is the primary treatment of dysmenorrhea, which includes oral contraceptive pills, systemic or local progestins, non-steroidal anti-inflammatory drugs, danazol and gonadotropin-releasing hormone analogues. Surgical therapies stay as the second line of treatment in refractory cases because medical therapies are associated with a failure rate of 20-25%^(3,4)^. Surgical intervention includes the interruption of a major group of cervical and uterine sensory nerve fibers, known as pelvic denervation. The presumed mechanism of presacral neurectomy (PSN) for pain relief of dysmenorrhea is primarily based on the anatomy of the sensory pathways from the pelvic viscera through the inferior and superior hypogastric plexus to the spinal column. Excision or incision of the superior hypogastric plexus, located in presacral area and so-called PSN, can disrupt many pain sensory pathways. The surgical technique of PSN was first described by Jaboulay^(5)^ and Ruggi^(6)^ as early as 1899. One hundred years after from the first description, Perez^(7)^ reported the first case of laparoscopic PSN (LPSN).

LPSN is found to be an effective surgical procedure in most cases refractory to medical treatment^(7,8,9,10)^; however, some patients still can not benefit from this approach. This diverse clinical response to LPSN has not yet been elucidated. One explanation may be that other sensory pathways not included in the superior hypogastric plexus may be responsible for cases with partial or no pain attenuation. Another possible mechanism is that the amount of excised neural tissue during LPSN may not be sufficient enough to resolve dysmenorrhea. Factors that determine the degree of response to PSN have yet to be enlightened. We hypothesized that there was an association between the amount of excised neural tissue and the response to therapy. The aim of the present study was to assess the correlation between the number of excised neural fibers and the degree of postoperative pain relief following LPSN.

## MATERIALS AND METHODS

Between July 2013 and August 2015, patients with the sole symptom of dysmenorrhea (pelvic pain during menstrual periods) for more than 6 months were consecutively enrolled in the study. All patients underwent multidisciplinary evaluation by the urology, gastroenterology, physical therapy, and psychiatry departments to exclude other potential causes of pelvic pain. All patients were questioned and physically examined to determine the exact location of their pain. Patients with pelvic pain related to specifiable pathologic conditions such as psychiatric disorder, malignant or infectious disease, previous pelvic surgery, large uterine leiomyoma, diagnosis of visually or histologically confirmed endometriosis or adenomyosis, and/or patients with lateral pelvic pain were excluded.

The study population consisted of 20 patients, whose most prominent symptoms were severe midline dysmenorrhea [Visual Analogue Scale (VAS) >80 mm] that had been unresponsive to at least two alternate medical therapies for more than 6 months. All patients reported job or school absenteeism in each menstrual period.

**Figure 1:**
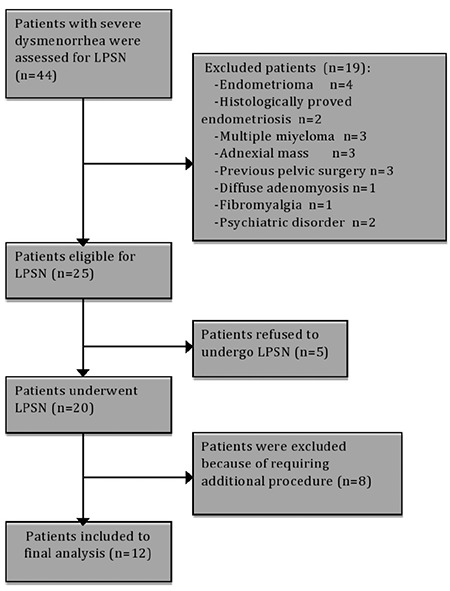
Flow chart of patients who were assessed for laparoscopic presacral neurectomy LPSN: Laparoscopic presacral neurectomy

Six of the 20 patients reported dyspareunia (painful sexual intercourse) and three had mild chromic pelvic pain (CPP) (pain that occurs below the umbilicus and lasts for at least six months) along with severe midline dysmenorrhea. Patients assessed for LPSN are summarized in a flow chart (Figure 1). Twenty patients underwent LPSN; however, during the laparoscopy, eight patients required additional endometriosis ablation or excision. To rule out the effect of additional procedures on postoperative pain levels, these patients were excluded from the final analysis.

Prior to the operation, all patients gave written informed consent for the details of surgical procedure including the risks, benefits, and recurrence rate of LPSN. A senior surgeon (M.A Massachusetts) performed all LPSN procedures with no intraoperative complications.

The severity of dysmenorrhea was assessed using a 100 mm VAS that ranged from “least possible pain” to “worst possible pain” at the time of hospital admission, and 2, 3, 6, and 12 months following the surgery. At admission, the women were requested to grade the most severe menstrual pain they experienced during the last 6 months using the VAS. The durations of the surgical procedures and hospital stay, blood loss, and intraoperative and postoperative complications were also recorded. All patients were followed up with clinical visits over 12 months to evaluate the degree of pain relief and possible complications. Zeynep Kamil Training and Research Hospital Review Board was approved our study. Written informed consent was provided from all patients to share and publish their medical records.

### Surgical procedure

In the steep Trendelenburg position, the small intestines and sigmoid colon over the sacral promontory were pushed out laterally. After identifying the ureter on the right side, the peritoneum overlying the sacral promontory was incised transversally between the sigmoid mesentery on the left side and right ureter on the right side. The tissue on the sacral promontory, presumably the superior hypogastric plexus, was elevated as much to the lateral sides as possible, carefully dissected from the areolar tissue and then, excised approximately 1 cm above and below the L5-S1 disc level using a harmonic scalpel (Ethicon Endo Surgery, Cincinnati, Ohio). The removed plexus segment was sent for histologic evaluation.

**Figure 2:**
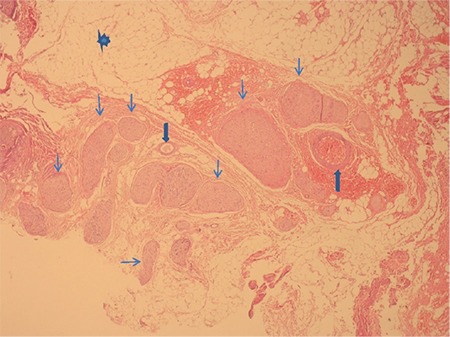
(Stained with hematoxylin and eosin, 40x) Neural fibers surrounded by adipose tissue. Neural fibers are indicated by thin arrows; vessels are shown by thick arrows, and the star demonstrates adipose tissue

Two pathologists (E.K. and H.Y.), who were blinded to the study interest, counted the neural fibers. The nerve specimens, after routine tissue processing, were cut into 3-μm-thick transverse, oblique, and longitudinal serial sections and deparaffinized in xylol for 10 minutes. They were dehydrated in a graded series of alcohol. The sections were kept in hematoxylin for 5-6 min. and washed with water for 5-10 min. They were then kept in eosin for 3-4 min and cleared in graded series of alcohol. After staining with hematoxylin-eosin, they were left to dry for a few minutes and placed in xylol. The tissues were passed through xylol three times for 10 min. Finally, they were taken out and mounted with a synthetic resin (entella). The sections were examined to determine the most appropriate sample, i.e., the section that included the greatest amount of neural fibers. To prevent repeated counting, the sections were mapped with a pencil (Figure 2).

### Statistical Analysis

Statistical analyses were performed using the SPSS software version 20 (SPSS, Inc., Chicago, Illinois, USA). While investigating the associations between non-normally distributed or ordinal variables, the correlation coefficients and their significance were calculated using the Spearman test. Friedman tests were used to compare the difference between the pre- and post-operative pain scores. In the post-hoc analysis of pairwise comparisons, the Wilcoxon test was performed. The agreement between the two pathologists for the neural fiber count was assessed using Lin’s concordance correlation coefficient (ρc). A 5% type 1 error level was used to infer statistical significance.

## RESULTS

Out of the initial 20 patients undergoing LPSN, eight were excluded from the final analysis due to an intraoperative diagnosis of endometriosis. All of these eight patients had dyspareunia and/or CPP along with dysmenorrhea. The remaining 12 patients were evaluated for the final analysis. The median age was 29 years. All patients had midline dysmenorrhea with a median (minimum-maximum) duration of 11 (7-15) years. The median body mass index was 24.6 kg/m2. The median (minimum-maximum) operation time for the LPSN was 32 (21-45) minutes. Blood loss was minimal. There were no minor or major vascular trauma, urinary or gastrointestinal complications related to LPSN. The patients were hospitalized for a maximum of 2 days postoperatively (median 1 day). There was no postoperative voiding dysfunction, constipation or other complications reported during the 12 months of follow-up.

Two pathologists reported the median (minimum-maximum) neural fiber count as 46 (20-85) and 47 (18-83). There was almost perfect agreement between the pathologists for the neural fiber counts [Lin’s concordance coefficient (pc)=0.99].

**Figure 3:**
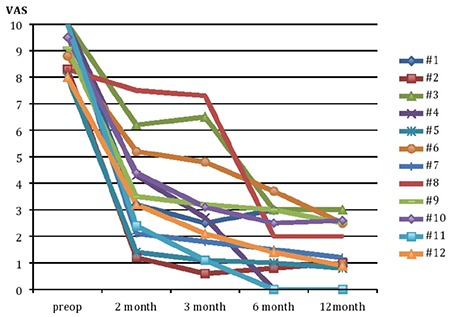
Visual Analogue Scale scores of 12 cases with dysmenorrhea before and 2nd, 3rd, 6th and 12th months after presacral neurectomy VAS: Visual Analogue Scale

The median pain scores were 9.2, 3.3, 2.6, 1.7, and 1.6 before, and 2, 3, 6, and 12 months following the operation, respectively. Pain scores significantly decreased at each follow-up visit compared with the preoperative period (p=0.002). The reduction of pain scores remained stable from 6 to 12 months (p=0.08) (Figure 3).

No statistically significant correlation was found between the neural fiber count and preoperative or postoperative pain scores (Table 1). After the procedure and throughout the follow-up period, all 12 patients reported no pain or mild pain without requiring pain medication.

## DISCUSSION

Up to 80% of reproductive aged women are affected by dysmenorrhea, in many cases causing sufficient pain that precludes social and occupational activities. Pain per se is a necessary defense mechanism of the body; however, chronic pain is a disturbing condition that needs to be treated. In persistent pain syndromes, people face distressing situations rather than biologic benefit. The presacral nerve, namely the superior hypogastric plexus, carries pain afferents from the cervix, the body of the uterus, and the proximal fallopian tube, but does not receive fibers from the ovaries and lateral pelvic structures. Therefore, PSN is traditionally performed for midline dysmenorrhea^(11)^. At this point, before deciding on surgical treatment, a detailed evaluation of patients to determine the location of pain has paramount importance in order to identify patients who would benefit from the procedure with the highest efficacy. In our study, we excluded subjects who reported lateral pain.

It has been shown that the success rate of PSN in the management of primary dysmenorrhea is 75 to 87%^(12,13,14)^. In the study by Jedrzejczak et al.^(13)^, the efficacy of LPSN was evaluated in patients with and without endometriosis. Dysmenorrhea decreased at 3 months by 75% in those without endometriosis. Furthermore, these patients reported a significant decline in dyspareunia and pelvic pain unrelated to menses at 3 and 12 months after LPSN. In our cases, the pain scores significantly decreased over months. The median pain scores decreased sharply at the 2nd month, gradually from 2nd to 3rd month and then, remained stable. The patients with dyspareunia and chronic pelvic pain reported substantial pain relief that did not require any analgesic medication. Although the number of our participants is limited to 12 cases, we obtained 100% of efficacy in patients with severe dysmenorrhea and no identifiable pelvic pathology, when nerve bundles were excised.

Previous studies reported that PSN might be ineffective in some cases. There is an on going debate as to whether efficacy could be related to the surgical method used. Some surgeons have asserted that a longer incision should be made on the hypogastric nerve plexus, whereas others claimed that the anatomic variation of nerve fibers at the area below the bifurcation of the aorta caused inconsistency in pain relief^(15)^. Furthermore, because the microscopic anatomy of a nerve plexus can not be differentiated during surgical exploration, surgeons only excise or transect the area where it is presumed to be the possible location of the superior hypogastric plexus. However, the architecture of the presacral area, which consists of fat, connective, vascular, and neural tissue, may mislead adequate excision of neural tissue. To ensure that the neural pathway has been included in the surgical area, histologic confirmation of the excised tissue is needed. On the other hand, the amount of removed neural tissue might be a determinant of surgical success. In our study, the number of neural fibers removed per specimen was counted by the pathologists. We detected that the neural fiber count differed from one patient to another and there was no correlation between the number of fibers removed per tissue and the amount of pain relief. This discrepancy can be explained by individual differences in the number of neural fibers or the complexity of pain perception. Moreover, the neural fiber intensity might not make any difference in pain transmission.

The method for PSN has been described in different approaches (laparotomy, laparoscopy, robotic) with different techniques (only transection or partial excision of nerve bundles) at different levels. Some preferred to excise a segment of 2-3 cm in diameter to prevent re-anastomosis of nerves, but others only transected without excision^(16,17)^. Chang et al.^(15)^ compared LPSN with modified LPSN by transecting nerve bundles over different levels to evaluate long-term effectiveness in pain relief. In traditional LPSN, the presacral nerve bundle is transected over the presacral promontory, whereas in the modified method, it is transected over the aortic bifurcation. Although pain relief was sustained for five years in 90% of patients with both methods, recurrent pain was reported by 82% of the patients in LPSN and 43% in modified LPSN eight years after the operations (p=0.04). The authors commented that incomplete resection and re-growth of the nerve fibers might cause this difference. Presacral nerves spread out into a latticework at the level of the first sacral vertebra, where it divides into several branches going to the right and left sides of the pelvis^(18)^. These nerves lie beneath fatty areolar tissue contained in the anterior longitudinal ligament, the middle sacral artery, and the vein plexus, where it is too difficult to distinguish and dissect them completely. In addition, there are individual variations of pelvic anatomy and neurophysiology, intermingling of afferent fibers, intercommunication among nerve plexuses, and cross-talk^(19)^. Excision of the nerves may keep these factors under control and contribute to the long-term effectiveness of the LPSN. We observed a reduction of pain in all subjects supporting the theory that segmental resection might increase the effectiveness of the procedure.

Our study sheds light on the physiology of pain transmission through the superior hypogastric plexus. However, to our knowledge, no studies have identified any association between the efficacy of the procedure and the quantitative or qualitative parameters of excised neural tissue. Inter-observer variability assessment between the pathologists provides the safety of this experimental nerve counting method, which makes it reproducible for further studies.

### Study Limitations

Our hypothesis was tested in a small number of cases. Further, the subtypes of the neural fibers were not differentiated.

## CONCLUSION

Our preliminary results revealed that LPSN, if properly performed in selected cases, is an effective surgical procedure to control primary dysmenorrhea. According to the results of our study, the degree of pain relief in severe midline dysmenorrhea is not related to the amount of excised neural tissue in LPSN. The pathophysiology of pain transmission and perception, and the underlying mechanism of the effectiveness of neurectomy need to be further elucidated.

## Figures and Tables

**Table 1:**
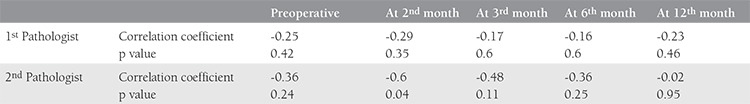
Correlation between the number of neural fibers evaluated by two pathologists and the pain scores at each interval
